# Bioenzymatic detoxification of mycotoxins

**DOI:** 10.3389/fmicb.2024.1434987

**Published:** 2024-07-18

**Authors:** Mengyu Liu, Xue Zhang, Haoni Luan, Yue Zhang, Wei Xu, Wei Feng, Peng Song

**Affiliations:** College of Life Sciences, Liaocheng University, Liaocheng, China

**Keywords:** mycotoxins, enzymes, detoxification, degradation, fungi, mechanism

## Abstract

Mycotoxins are secondary metabolites produced during the growth, storage, and transportation of crops contaminated by fungi and are physiologically toxic to humans and animals. Aflatoxin, zearalenone, deoxynivalenol, ochratoxin, patulin, and fumonisin are the most common mycotoxins and can cause liver and nervous system damage, immune system suppression, and produce carcinogenic effects in humans and animals that have consumed contaminated food. Physical, chemical, and biological methods are generally used to detoxify mycotoxins. Although physical methods, such as heat treatment, irradiation, and adsorption, are fast and simple, they have associated problems including incomplete detoxification, limited applicability, and cause changes in food characteristics (e.g., nutritive value, organoleptic properties, and palatability). Chemical detoxification methods, such as ammonification, ozonation, and peroxidation, pollute the environment and produce food safety risks. In contrast, bioenzymatic methods are advantageous as they achieve selective detoxification and are environmentally friendly and reusable; thus, these methods are the most promising options for the detoxification of mycotoxins. This paper reviews recent research progress on common mycotoxins and the enzymatic principles and mechanisms for their detoxification, analyzes the toxicity of the degradation products and describes the challenges faced by researchers in carrying out enzymatic detoxification. In addition, the application of enzymatic detoxification in food and feed is discussed and future directions for the development of enzymatic detoxification methods are proposed for future in-depth study of enzymatic detoxification methods.

## 1 Introduction

Mycotoxins are secondary metabolites produced by certain fungi that infect crops (grains, nuts, and fruits) and pose a threat to humans and animals (Xu et al., 2022). Humans are exposed to mycotoxin either by directly consuming mycotoxin-present crops or indirectly by ingesting through the consumption of livestock or poultry fed contaminated crops (Abraham et al., 2022a). Short-term consumption of highly mycotoxin-contaminated food/feed can be fatal to humans and animals, while prolonged exposure to threshold doses can result in liver and nervous system damage, immunosuppression and chronic diseases with long-term carcinogenic effects (Yu and Pedroso, 2023). At present, the detoxification of mycotoxins is commonly achieved by physical, chemical, and biological methods. Physical detoxification methods, such as thermal treatment, irradiation, and adsorption, are fast and simple, but may result in incomplete detoxification; however, their applicability is limited due to potential impacts on food characteristics (e.g., nutritive value, organoleptic properties, and palatability) (Haidukowski et al., 2019; Murugesan et al., 2021; Raesi et al., 2022; Zhang et al., 2022; Jing et al., 2023; Romero-Sánchez et al., 2024). Chemical detoxification methods, such as ozonation, peroxidation, and ammoniation achieve high detoxification efficiencies but are associated with environmental degradation, the generation of hazardous by-products, the use of potentially toxic reagents, and significant costs (Afsah-Hejri et al., 2020; Shen and Singh, 2022a,b; Demirci et al., 2023). Newly developed physicochemical detoxification methods, such as cold plasma technology (Gavahian and Khaneghah, 2020), pulsed light sterilization technology (Wang L. et al., 2022; Li et al., 2023b), and the application of novel nanomaterials (Lin et al., 2022), also affect food characteristics and are costly. Enzymatic detoxification involves the use of biological enzymes to reduce or remove mycotoxins. Compared with traditional physical and chemical detoxification methods, enzymatic detoxification is performed under mild conditions without nutrient loss or secondary contamination, while being highly efficient, versatile, highly specific, and low in toxicity.

**Figure 1:**
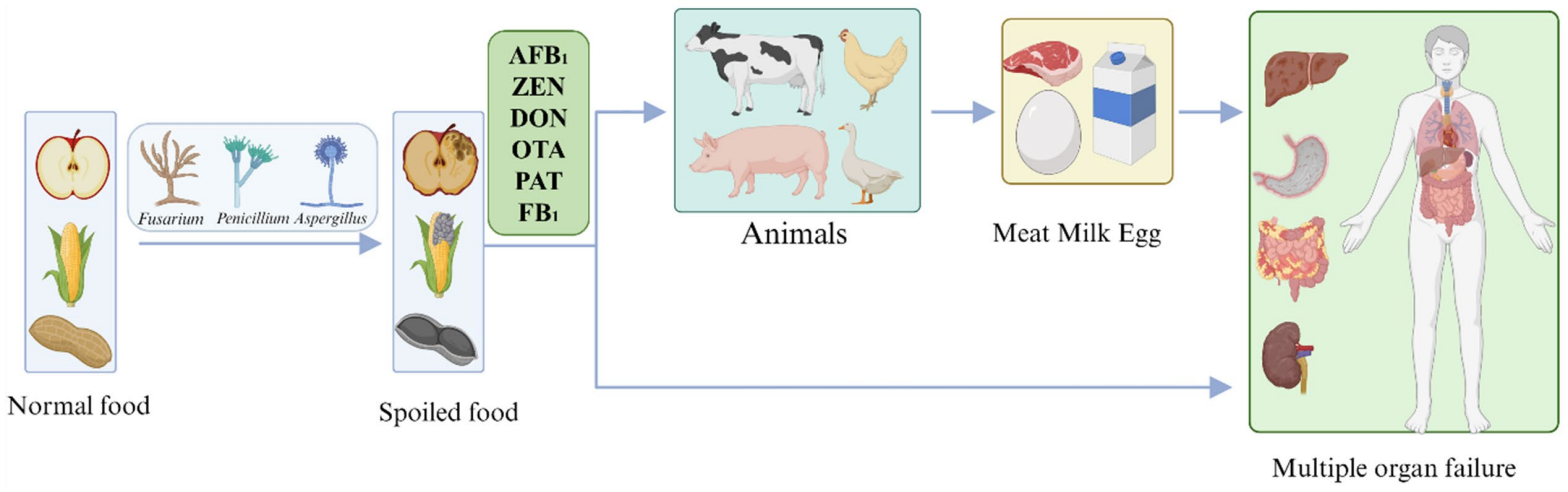
The flow direction of mycotoxins in foods. AFB_1_, Aflatoxin B_1_; ZEN, Zearalenone; DON, Deoxynivalenol; OTA, Ochratoxin; PAT, Patulin; and FB_1_, Fumonisin B_1_.

To date, more than 400 mycotoxins have been identified that belong to different categories and originate from different fungi, the most common of which are aflatoxin, zearalenone deoxynivalenol, ochratoxin, patulin, and fumonisin. Mycotoxins not only contaminate food production systems, causing enormous losses to the global economy, but are also passed on to humans through the food chain, posing a major threat to human health. The directional flow direction of mycotoxin contaminants in food is shown in Figure 1. Complete elimination becomes a challenge once mycotoxins are present. The most effective approach to preventing mycotoxin contamination is to intervene before fungal infections occur and before plants produce mycotoxins (Jouany, 2007).

Cultivate healthy seeds of disease- and pest-resistant varieties that can resist fungal diseases and prevent fungal invasion. Use crop rotation, appropriate irrigation and fertilization, fungicides and disinfection are used during the growing season to reduce the population of toxigenic fungi. Timely harvesting of mature crops, prompt drying to control moisture content, and storage and transport in a dry environment. Protecting the external structure of seeds and grains minimizes the risk of fungal contamination. High-risk components of mycotoxins can be removed by removing diseased plants, discarding damaged grains, and thoroughly washing grain soil (Jouany, 2007; Ogunade et al., 2018; Matumba et al., 2021; Nada et al., 2022). Governments can use publicity and education to raise people’s awareness of the toxic hazards of mycotoxins and to encourage good hygiene habits in their daily lives. Countries should also implement stricter guidelines for mycotoxin levels in cereals and develop relevant legislation policies to minimize the threat of mycotoxins to humans.

To our knowledge, existing reviews do not comprehensively evaluate the types of mycotoxin detoxification enzymes or their degradation mechanisms. And only a few reviews have been published to date to systematize them (Ben Taheur et al., 2019; Lyagin and Efremenko, 2019; Venkatesh and Keller, 2019; Abdi et al., 2021; Abraham et al., 2022a; Wang Y. et al., 2022; Sun H. et al., 2023). This review provides a detailed summary of the principles and mechanisms underlying the bioenzymatic detoxification of mycotoxins that are prevalent in major crops, analyzes the toxicity of the degradation products, and discusses their potential application in the detoxification of mycotoxins in food and feed. Through this review, it is hoped that the understanding of mycotoxin detoxification enzymes will be more thorough and will provide a theoretical and practical basis for future research and applications related to the biological detoxification of mycotoxins in the food and feed industries.

## 2 Common mycotoxins and corresponding enzymes used for detoxification

### 2.1 Aflatoxins

**Figure 2:**
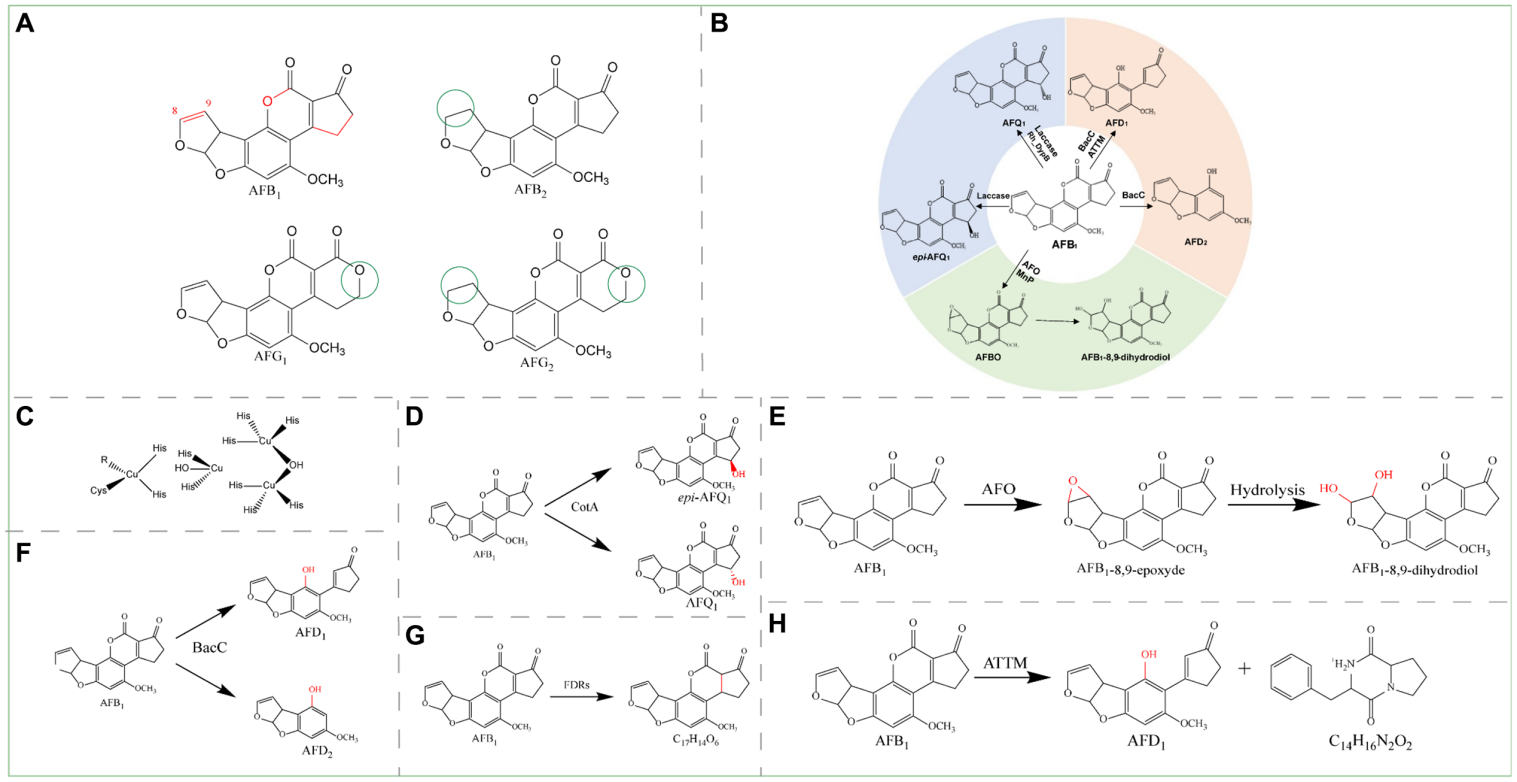
**(A)** Structural formula of AFB_1_ and its derivatives. The red group is the detoxifying enzyme action group and the green group shows the difference between AFB_1_ derivatives and them. AFB_1_, Aflatoxin B_1_; AFB_2_, Aflatoxin B_2_; AFG_1_, Aflatoxin G_1_; AFG_2_, Aflatoxin G_2_. **(B)** Main degradation and transformation products of AFB_1_. **(C)** Structure of the active center of laccase. **(D)** Detoxification of AFB_1_ by CotA laccase. **(E)** Degradation of AFB_1_ by AFO. **(F)** Degradation of AFB_1_ by BacC. **(G)** Degradation of AFB_1_ by FDRs. **(H)** Degradation of AFB_1_ by ATTM.

Aflatoxin (AF), a derivative of difurocoumarin, is produced by *Aspergillus* fungi and is typically found in corn, peanuts, rice, nuts, and other crops infected with *Aspergillus flavus*; these infections result from long-term storage, poor storage conditions, and poor sanitation during processing (Aristil et al., 2020). Among the various types of aflatoxins, the most common are aflatoxin B_1_ (AFB_1_), aflatoxin B_2_ (AFB_2_), aflatoxin G_1_ (AFG_1_), and aflatoxin G_2_ (AFG_2_) (Figure 2A; Du et al., 2023).

Aflatoxin B_1_ is the most common, most toxic, and most thoroughly researched class of mycotoxins and was classified by the World Health Organization as a group 1 carcinogen in 1993 (Janik et al., 2020). Liver cancer is likely to occur after long-term exposure to AFB_1_ (Do et al., 2020), and 28% of liver cancer cases worldwide are caused by AFB_1_ (Liu and Wu, 2010; Liu et al., 2012). For food safety reasons, more than 100 countries and regions have established detailed limit standards for different types of food. For example, the European Union (EU) has set a limit of 2 μg/kg AFB_1_ for all cereals and cereal products (Commission Regulation (EU), 2010). The mechanism by which AFB_1_ causes liver cancer involves the oxidation of AFB_1_ in the liver by cytochrome P450 3A4 (CYP3A4) in microsomes; this process generates the reactive metabolite AFB_1_-8,9-epoxide (AFBO), which covalently binds to nucleic acids and leads to liver cancer. A study in poultry revealed that the toxin is metabolized mainly by cytochrome P450 1A2 (CYP1A2) and P450 3A4 (CYP3A4) and causes numerous mutations, particularly in the p53 tumor suppressor gene (Ülger et al., 2020). The main toxic groups of AFB_1_ include the C8-C9 double bond on the difuran ring, the lactone ring in the coumarin structure, and the pentanone ring (Figure 2A). There are three main pathways by which toxic groups can be targeted to degrade AFB_1_: the first pathway involves the C8-C9 double bond on the difuran ring in the toxin structure, the second pathway involves lactone ring modification or ring opening, and the third pathway involves hydroxylation and reduction of the pentenone ring. The main degradation and transformation products of AFB_1_ include aflatoxin Q_1_ (AFQ_1_), *epi*-aflatoxin Q_1_ (*epi*-AFQ_1_), aflatoxin D_1_ (AFD_1_), aflatoxin D_2_ (AFD_2_), and AFBO. Due to the instability of AFBO, AFB_1_-8,9-dihydrodiol is eventually generated (Figure 2B). AFB_1_ toxicity can be reduced by the epoxidation of C8-C9, hydroxylation of C3, or epoxidation and modification of the lactone ring. Oxidoreductases, lactonucleases, and other degrading enzymes can catalyze the conversion of these groups, thereby achieving enzymatic detoxification.

#### 2.1.1 Oxidoreductase

Oxidoreductases, such as laccases, oxidases, reductases, and peroxidases, degrade AFB_1_ by different mechanisms. Degradation of AFB_1_ by laccases involves hydroxylation of the pentanone ring, aflatoxin oxidase involves oxidation of the furan ring, reductases involve reduction of the double bond of the coumarin lactone ring and the α, β-unsaturated ester bond between the lactone ring and the pentanone ring, and peroxidases involve hydroxylation of the pentanone ring and oxidation of the furan ring by mechanisms similar to those of laccase and aflatoxin oxidase, respectively.

##### 2.1.1.1 Laccase

Laccases are a very important class of oxidoreductases that can degrade AFB_1_. As an extracellular multicopper oxidase, laccase can catalyze the oxidation of aromatic amines, phenols, carboxylic acids, and other heterocyclic compounds to less toxic or nontoxic compounds; the structure of its active center is shown in Figure 2C. Laccase can oxidize AFB_1_ indirectly through a mediator or directly (Okwara et al., 2021); in practical applications, direct oxidation is a more feasible route. In indirect oxidation, the mediator is first oxidized by laccase, and then the oxidized mediator can oxidize the substrate. Through this system, laccases can overcome the limitation of redox potential and expand the range of substrates. However, copper clusters in laccases directly oxidize the substrate through free radicals generated during direct oxidation.

With acetosyringone (AS) as the mediator, Ery4 laccase can completely remove 0.1 μg/mL AFB_1_ *in vitro* after 1 h and likely converts the compounds into AFQ_1_, *epi*-AFQ_1_, AFB_1_-8,9-dihydrodiol or AFB_1_-dialdehyde, AFB_2a_, and AFM_1_ (Loi et al., 2023). With methyl syringate (MS) as a mediator, the *Bs*CotA laccase from *Bacillus subtilis* oxidizes AFB_1_ to AFQ_1_; in this process, the toxicity of the product is significantly reduced, and the conversion efficiency depends on the dosages of laccase and the mediator (Wang et al., 2024). In another experiment, CotA laccase from *Bacillus licheniformis* ANSB821 was expressed in *E. coli*, AFB_1_ was directly converted to AFQ_1_ and *epi*-AFQ_1_ effectively in the absence of redox mediators (Figure 2D), and the degradation rate reached 96% within 12 h at pH 8.0 and 37°C (Guo et al., 2020). Compared with CotA laccase, the Q441A mutant exhibited better pH stability, better thermal stability and higher degradation efficiency (Liu et al., 2023a). The recombinant laccase Lac3 from *Trametes* sp. C30 broke the double bond of the furan ring and lactone ring in the coumarin moiety and degraded AFB_1_ into C_16_H_22_O_4_, C_14_H_16_N_2_O_2_, C_7_H_12_N_6_O, and C_24_H_30_O_6_ with a degradation rate of 91%; in addition, the toxicity of the degradation products was significantly reduced. Studies in mouse HepG2 cells have shown that Lac3-degraded AFB_1_ reduces hepatocyte apoptosis, histopathological lesions in the liver and kidney, oxidative stress, and inflammation (Liu et al., 2021). Recombinant fmb-rL103 laccase (2.5 mM) from *Bacillus vallismortis* can degrade AFB_1_ (10 ng/mL) at 37°C and pH 7.0 with a degradation rate greater than 50% (Bian et al., 2022). However, the degradation mechanism remains uncertain.

##### 2.1.1.2 Oxidase

Aflatoxin oxidase (AFO) was the first enzyme discovered that could degrade AFB_1_ (Cao et al., 2011). AFO has no obvious similarity to any known oxidase, and its protein structure suggests that it is a new aflatoxin oxidase that is different from reported oxidases such as laccase; it is therefore listed separately. AFO is an intracellular enzyme that acts on the AFB_1_ difuran ring and oxidizes it to AFBO. Due to the instability of AFBO, AFBO is further hydrolyzed to AFB_1_-8,9-dihydrodiol (Figure 2E) (Wu et al., 2015).

##### 2.1.1.3 Reductase

The reductase BacC from *Bacillus subtilis* UTB1, which catalyzes the degradation of AFB_1_, may catalyze the reduction of the double bond of the lactone ring within the coumarin moiety. The ester bond then undergoes hydrolysis to yield carboxylic acid, and AFD_1_ is produced through decarboxylation. Alternatively, after hydrolysis, the derivative AFD_2_ can be produced through cleavage of the bond with the ciclopeptone ring (Figure 2F). BacC contributes to the reduction of aflatoxin levels in pistachio fruits: treatment of pistachio nuts with *B. subtilis* UTB1 incubated at 30°C for 7 days reduced aflatoxin levels by 69.1% (Afsharmanesh et al., 2018).

F_420_H_2_-Dependent reductases A and B (FDR-A and FDR-B) from *Mycobacterium smegmatis* were previously found to catalyze the reduction of α, β-unsaturated ester bonds between the lactone and pentanone rings (Figure 2G) (Taylor et al., 2010). Subsequently, it was found that MSMEG_5998 from the FDR family of *Mycobacterium smegmae* exhibits greater degradation activity toward AFB_1_ in the pH range of 7.0–10.0, and compared with native enzymes, the thioredoxin Trx-linked MSMEG_5998 recombinant enzyme exhibits greater degradation activity *in vitro*. In a HepG2 cell culture model, Trx-linked MSMEG_5998 can reduce AFB_1_-induced cytotoxicity in HepG2 cells by ameliorating DNA damage and p53-mediated apoptosis (Li et al., 2019).

##### 2.1.1.4 Peroxidase

Heterologous expression with the manganese peroxidase PhcMnp in *Kluyveromyces lactis* generated a product that exhibited a good ability to degrade AFB_1_. The degradation rate reached 75.71% at 40°C and pH 4.5 for 40 h, and the degradation product was identified as AFB_1_-8,9-dihydrodiol. Peanut samples contaminated with AFB_1_ were treated, and the recombinant strain degraded more than 90% of the AFB_1_ in the peanut samples (Xia et al., 2022).

The recombinant type B dye decolorizing peroxidase (Rh_DypB) can degrade AFB_1_ to AFQ_1_ and numerous low molecular mass compounds *in vitro*. After reacting with 0.1 U/mL Rh_DypB and 0.1 mM H_2_O_2_ for 96 h, the degradation rate of AFB_1_ reached 96% (Loi et al., 2020). The dye decolorizing peroxidase type B (DypB) can effectively degrade AFB_1_ under the action of Mn^2+^ (Mangini et al., 2024).

Ery4 laccase cannot degrade AFB_1_ without a redox mediator; in contrast, MnP requires H_2_O_2_, and Dyp requires H_2_O_2_ and Mn^2+^ as cofactors to degrade AFB_1_. However, due to the potential toxicity and high cost of the redox mediators, H_2_O_2_ and Mn^2+^, these mechanisms are not practical for food and feed applications. Therefore, laccases without redox mediators, such as CotA laccase, Lac3 laccase, and fmb-103 laccase, and oxidoreductases without cofactors are advantageous for the detoxification of mycotoxins in food and feed.

#### 2.1.2 Lactonase

Lactonases are a family of enzymes that degrade AFB_1_ by destroying the AFB_1_ lactone ring, and opening the lactone ring can effectively reduce the toxicity of AFB_1_. Certain *Bacillus* species possess the aiiA gene, which encodes N-acyl-homoserine lactone (AHL) lactonase; this lactonase is responsible for AFB_1_ degradation and cleaves the lactone ring by targeting the lactone bond. This enzymatic hydrolysis mechanism is based on an AHL with a structure similar to that of AFB_1_, which also possesses a lactone ring. In a toxicity test with *Artemia salina*, these *Bacillus* strains were able to significantly reduce the toxicity of AFB_1_ after incubation at 30°C for 48 h (González Pereyra et al., 2019). The highest rate of AFB_1_ degradation by ATTM lactonase from *Bacillus megaterium* HNGDA6 was 86.78% at 80°C and pH 8.5, and the degradation products were AFD_1_ (C_16_H_14_O_5_) and C_14_H_16_N_2_O_2_. The former product results from the decarboxylation of the lactone ring-opening form of AFB_1_, whereas the other three toxicity sites (the C8-C9 double bond of the difuran ring, the lactone ring, and the pentanone ring) are disrupted (Figure 2H) (Cheng et al., 2023).

#### 2.1.3 Degrading enzyme

*Pantoea* aflatoxin degradation enzyme (PADE_1_) is an AFB_1_-degrading enzyme from *Pantoea* sp. T6 that was identified as an outer membrane protein A and showed the highest degradation rate of AFB_1_ (48.5%) at 40°C and pH 7.0 (Xie et al., 2019). However, the *Pseudomonas* AFB_1_-degrading enzyme (PADE_2_) from *Pseudomonas aeruginosa* M19 showed the greatest AFB_1_ degrading activity at 65°C and pH 6.0. Cu^2+^ and Fe^3+^ strongly enhanced the activity, whereas Ca^2+^ and Zn^2+^ strongly inhibited the activity (Song et al., 2019). The genetically engineered *Trametes versicolor* AFB_1_-degrading enzyme (TV-AFB_1_D) converted 67.4% of AFB_1_ to other compounds within 5 h at 32°C and pH 7.0. Recombinant TV-AFB_1_D engineered *Saccharomyces cerevisiae* strains effectively degrade AFB_1_ in contaminated rice (Yang P. et al., 2022).

The above three types of enzymes (oxidoreductases, lactonases, and degrading enzymes) can degrade AFB_1_, but only a few studies have investigated the degradation and transformation mechanism of enzymes and identified the structure of degradation products; some intermediate degradation products and the catalytic mechanisms of enzymes remain unknown. More detoxification enzymes and degradation products should be identified to clarify the biological detoxification mechanisms of these enzymes. The detoxifying enzymes of AFB_1_ are summarized in Table 1.

**Table 1:** The detoxifying enzymes of AFB_1_.

Enzymes	Sources	Category	Degradation product(s)	Toxicity	References
Ery4	*Saccharomyces cerevisiae* ITEM 17289	Laccase	AFQ_1_, *epi*-AFQ_1_, AFB_2a_, AFM_1_, AFB_1_-8,9-dihydrodiol or AFB_1_-dialehyde	Reduce	Loi et al. (2023)
*Bs*CotA	*Bacillus subtilis*	Laccase	AFQ_1_	Reduce	Wang et al. (2024)
CotA	*Bacillus licheniformis* ANSB821	Laccase	AFQ_1_, *epi*-AFQ_1_	Reduce	Guo et al. (2020)
CotA-Q441A	*Bacillus subtilis*	Laccase	AFQ_1_, *epi*-AFQ_1_	Reduce	Liu et al. (2023a)
Lac3	*Trametes* sp. *C30*	Laccase	C_16_H_22_O_4_, C_14_H_16_N_2_O_2_, C_7_H_12_N_6_O, and C_24_H_30_O_6_	Reduce	Liu et al. (2021)
fmb-103	*Bacillus vallismortis* fmb-103	Laccase	Unknown	Unknown	Bian et al. (2022)
AFO	*Armillariella tabescens*	Oxidase	AFB_1_-8,9-dihydrodiol	Reduce	Cao et al. (2011); Wu et al. (2015)
BacC	*Bacillus subtilis* UTB1	Reductase	AFD_1_, AFD_2_	Reduce	Afsharmanesh et al. (2018)
FDR	*Mycobacterium smegmatis*	F_420_H_2_ dependent reductases	C_17_H_14_O_6_	Reduce	Taylor et al. (2010); Li et al. (2019)
pKLAC1-Phc*mnp*	*Kluyveromyces lactis strains* GG799	Manganese peroxidase	AFB_1_-8,9-dihydrodiol	Reduce	Xia et al. (2022)
Rh_DypB	Unknown	Dye decolorizing peroxidase	AFQ_1_	Reduce	Loi et al. (2020)
DypB	Unknown	Dye decolorizing peroxidase	C_17_H_14_O_6_, C_16_H_14_O_6_, C_16_H_14_O, C_17_H_14_O_7_	Unknown	Mangini et al. (2024)
AHL	*Bacillus*	Lactonase	Unknown	Reduce	González Pereyra et al. (2019)
ATTM	*Bacillus megaterium* HNGD-A6	Lactonase	AFD_1_, C_14_H_16_N_2_O_2_	Reduce	Cheng et al. (2023)
PADE_1_	*Pantoea* sp. T6	Unknown	Unknown	Unknown	Xie et al. (2019)
PADE_2_	*Pseudomonas aeruginosa* M19	Unknown	Unknown	Unknown	Song et al. (2019)
TV-AFB_1_D	*Trametes versicolor*	Unknown	Unknown	Unknown	Yang P. et al. (2022)

### 2.2 Zearalenone

**Figure 3:**
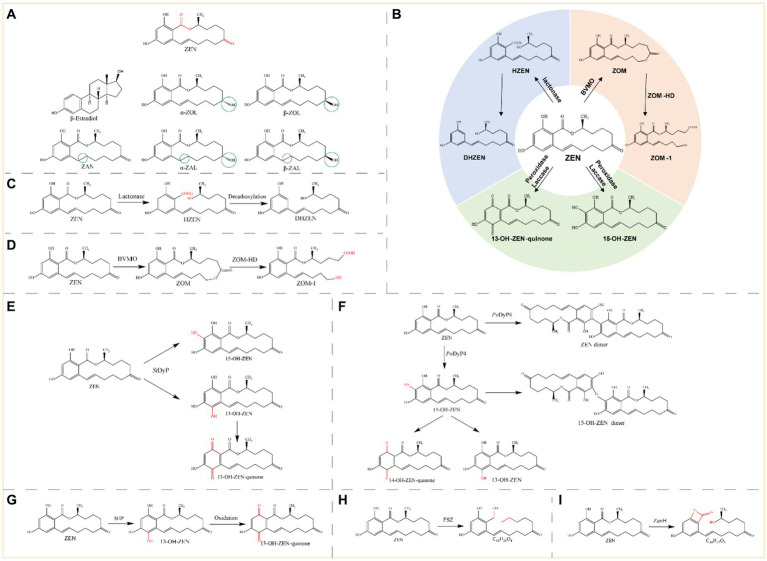
**(A)** Structural formula of ZEN and its derivatives. The red group is the detoxifying enzyme action group and the green group shows the difference between ZEN derivatives and them. ZEN, Zearalenone; β-estradiol; α-ZOL, α-Zearalenol; β-ZOL, β-Zearalenol; ZAN, Zearalanone; α-ZAL, α-Zearalanol; β-ZAL, β-Zearalanol. **(B)** Main degradation and transformation products of ZEN. **(C)** Degradation of ZEN by lactonase. **(D)** Degradation by monooxygenase and carboxylester hydrolase. **(E)** Degradation of ZEN by *St*DyP. **(F)** Degradation of ZEN by *Po*DyP4. **(G)** Degradation of ZEN by SHP. **(H)** Degradation of ZEN by FSZ. **(I)** Degradation of ZEN by ZenH.

Zearalenone (ZEN) is a toxic compound with a resorcylic acid lactone structure produced by *Fusarium* fungi (Figure 3A). This toxin is present in moldy grains, such as barley, corn, and wheat, and typically results from improper postharvest storage or processing (Bouajila et al., 2022). ZEN is a non-steroidal estrogen mycotoxin that interferes with the endocrine system and exerts toxic effects on the reproductive system of animals. ZEN can cause adverse conditions such as non-infectious abortion, an abnormal estrous cycle, and abnormal embryo development (Yang et al., 2020; Yadav et al., 2021). ZEN has five structural derivatives: α/β-zearalenol (α/β-ZOL), α/β-zearalanol (α/β-ZAL), and zearalanone (ZAN) (Figure 3A). ZEN and its derivatives possess chemical structures similar to those of the animal endogenous estrogen β-estradiol; thus ZEN and its derivatives exhibit toxicity through interactions with the receptors of β-estradiol in animal cells, and compared with ZEN, α-ZAL, and α-ZOL exhibit greater cytotoxicity (Jouany, 2007).

Therefore, although ZEN is the most abundant toxin in moldy grains, the toxicity of both the ZEN molecule and its derivatives should be emphasized. The main toxic group of ZEN is the lactone ring (Figure 3A), and the known pathways of ZEN biodegradation involve hydrolysis of the lactone ring, oxidation of the ketone group (or hydrolysis followed by the addition of oxygen), and hydroxylation of the aromatic groups. The main degradation products of ZEN include DHZEN, ZOM-1, 15-OH-ZEN, and 13-OH-ZEN-quinone (Figure 3B). Lactonases can hydrolyze the ester bond on the lactone ring of zearalenone, and the toxicity of the hydrolyzed product is significantly reduced or even eliminated. Monooxygenases and carboxylester hydrolases destroy the C6 ketone group of ZEN, and the metabolite ZOM-1 loses its estrogenic activity. Peroxidases and laccases can hydroxylate the aromatic ring on ZEN, the resulting products are transformed into 13-OH-ZEN-quinone and 15-OH-ZEN, respectively, and the estrogenic biotoxicity of both products is significantly reduced. Lactonases, oxidoreductases and other novel enzymes detoxify ZEN by different mechanisms, generating different products.

#### 2.2.1 Lactonase

Zearalenone lactone hydrolase (ZHD) degrades ZEN by disrupting the ZEN lactone structure, thereby achieving ZEN detoxification. Lactonase first hydrolyses ZEN to hydrolyzed ZEN (HZEN), which is further decarboxylated to form decarboxylated HZEN (DHZEN) (Figure 3C).

Takahashi-Ando et al. (2002) cloned and expressed a lactonohydrolase from *Clonostachys rosea* IFO 7063 in *Escherichia coli* and were the first to report the mechanism by which the lactonohydrolase ZHD101 promotes ZEN degradation and detoxification. The ZEN hydrolase RmZHD from *Rhinocladiella mackenziei* degraded ZEN through proton transfer and nucleophilic-substituted ring opening to form a hydroxyl product (Zhou et al., 2020). The amino acid sequences of ZLHY-6 from *Clonostachys rosea* 31535 and ZHD101 exhibited high identity (98.3%). The optimum temperature and pH ranges for ZLHY-6 to ZEN were 25–41°C and 6.5–10, respectively. By optimizing ZLHY-6, irreversible degradation of ZEN was achieved, with a degradation rate of up to 96.31%. The ZEN in crude oil was reduced from 1257.3 to 13 μg/kg after detoxification with ZLHY-6 during crude oil refining (Chang et al., 2020). PR-ZHD from *Clonostachys rosea* GRZ7 was successfully heterologously expressed in *Penicillium canescens* strain PCA-10, and ZEN was completely removed from the model solutions after 3 h at pH 8.5 and 30°C. The addition of PR-ZHD (8 mg/g of dried grain) to flour samples prepared from the infected ZEN contaminated grain (approximately 16 μg/g) completely decontaminated the samples after an overnight exposure (Shcherbakova et al., 2020). The BAMF_RS30125 gene from *Bacillus amyloliquefaciens* H6 was heterologously expressed in *E. coli*, and the recombinant purified protein ZTE138 was obtained. The degradation rates of ZEN were 34.20 and 59.79% at 36 and 72 h, respectively (Xu et al., 2020). ZHD607 from *Phialophora americana*, a mesophilic lactone hydrolase, was heterologously expressed in *Pichia pastoris* and showed optimum activity at 35°C and pH 8.0. Two ZHD607 mutants, ZHDM1 and I160Y, showed 2.9- and 3.4-fold greater ZEN degradation, respectively, than did ZHD607 (Yu et al., 2020). ZENG from *Gliocladium roseum* MA918 was identified as a highly efficient degrading enzyme. The recombinant ZENG showed maximum activity at pH 7.0 and 38°C (> 70% degradation at ZEN concentrations of 10–80 μg/mL). This is the first report of a neutral ZEN-degrading lactonase that can degrade ZEN and its derivatives (α-ZOL and α-ZAL) (Zhang et al., 2020). In addition, the half-life (*t_1/2_*) of the thermostable ZENG mutant S162P/S220R was 36.8-fold greater than that of the wild-type enzyme at 55°C, and the melting temperature (*T*m) was 8.2°C higher (Fang et al., 2022). ZenA showed high degradation activity for ZEN, converting ZEN to HZEN within the first 10 min of the reaction, and HZEN was partially decarboxylated to DHZEN during the 2 h of the reaction. The metabolism of ZEN in the reticulorumen of dairy cows was studied and the concentration of ZEN in the reticulorumen was significantly reduced by ZenA administration (Gruber-Dorninger et al., 2021). ZHD-P from *Trichoderma aggressivum* exhibited 97% amino acid sequence identity with ZHD101, and the specific activity of the heterologously expressed and purified ZHD-P was 1.55-fold greater than that of ZHD101. The optimum temperature and pH for ZEN degradation were 45°C and 7.5–9.0, respectively, and the degradation activity of ZHD-P increased with the addition of Ca^2+^, K^+^, Mg^2+^, Mn^2+^, and Na^+^ (Chen S. et al., 2021). ZHD_LD from *Exophiala spinifera* exhibited 60.15% amino acid identity with ZHD101, and the purified recombinant ZHD_LD showed high ZEN degradation activity under optimum reaction conditions (50°C and pH 9.0) (Gao et al., 2022). The novel lactonase Zhd11B from *Fonsecaea monophora* is a neutral lactone hydrolase with a broad substrate spectrum that can efficiently hydrolyze ZEN and its more toxic derivatives (α-ZAL and β-ZAL). Through cap domain swapping, the activities of ZEN, α-ZAL and β-ZAL were further improved by 1.5-, 1.6-, and 2.9-fold, respectively (Jiang et al., 2022). Zhd11D from *Phialophora attinorum*, which has the highest degradation rate of ZEN at pH 8.0 and 35°C, increased enzyme activity by 1.5-fold and thermostability by 2-fold (40°C) by fusing a segment of the amphiphilic short peptide S1 at the N-terminus of Zhd11D. In addition, S1-Zhd11D showed a higher rate of hydrolysis of ZEN than Zhd11D in peanut oil (Wang Z. et al., 2023).

Most zearalenone-degrading lactonases are temperature sensitive and can only maintain high activity within a narrow temperature range. Therefore, resolving the structure of lactonases and improving their temperature stability represent future research directions.

#### 2.2.2 Oxidoreductase

Monooxygenases, peroxidases, and laccases are oxidoreductases that degrade ZEN. Baeyer-Villiger monooxygenases (BVMOs) and carboxylester hydrolases (ZOM-HDs) from *Apiotrichum mycotoxinivorans* function together to achieve detoxification (Mascotti et al., 2015). First, a BVMO enzyme catalyzes the insertion of an oxygen atom between C5 and C6, thus transforming the cyclic ketone to a zearalenone oxidized metabolite (ZOM). Next, ZOM-HD is recruited to hydrolyze ZOM, breaking the ester bond at C6 to produce a non-estrogen compound (ZOM-1) (Figure 3D) (Sun et al., 2020).

The *St*DyP gene from *Streptomyces thermocarboxydus* was expressed in *E. coli*, and *St*DyP was able to slightly degrade ZEN; the process could be accelerated in the presence of Mn^2+^ and 1-hydroxybenzotriazole, and the degradation products were 15-OH-ZEN and 13-OH-ZEN. However, 13-OH-ZEN is unstable and autooxidizes to 13-OH-ZEN-quinone (Figure 3E) (Qin et al., 2021b).

The spore CotA laccase from *Bacillus licheniformis* was heterologously expressed in *E. coli* and could directly oxidize ZEN; moreover, the addition of the redox mediators 2,2′-azino-bis-(3-ethylbenzothiazoline-6-sulphonic acid) (ABTS) and AS promoted the degradation of ZEN by the CotA laccase. Proliferation assays in human breast cancer MCF-7 cells showed that CotA laccase and CotA laccase-mediator systems eliminated the estrogenicity of ZEN. Additionally, the immobilization of CotA laccase onto chitosan microspheres resulted in greater thermal stability than that of free CotA laccase. The rates of ZEN removal in corn meal by free and immobilized CotA laccase were 70 and 90%, respectively (Guo et al., 2022).

*Po*DyP4, a dye-decolorizing peroxidase from *Pleurotus ostreatus*, was expressed in *E. coli*. It almost completely degraded 1 mM ZEN within 2 h at pH 6.0 and 40°C with good pH and temperature stability. ZEN can be detoxified by hydroxylation, oxidation, and polymerization reactions (Figure 3F) (Ding et al., 2024).

Soybean hull peroxidase (SHP) extracted from soybean hulls degraded 95% of the ZEN in 100 μM H_2_O_2_ buffer when added in a stepwise manner within 1 h. The degradation of ZEN by SHP in maize steep liquor, maize flour, wheat flour, and rice flour was 85, 60, 66, and 54%, respectively, (Figure 3G) (Guo et al., 2024).

#### 2.2.3 Novel enzymes for ZEN degradation

Several novel ZEN-degrading enzymes have also been identified. The ZEN-degrading enzyme FSZ from *Aspergillus niger* ZEN-S-FS10 is a novel enzyme (with less than 10% homology to ZHD101 and RmZHD) that degrades ZEN at a rate of 75–80% (pH 7.0, 28°C) and produces C_18_H_26_O_4_ as the degradation product (Figure 3H). FSZ can function at 28–38°C and pH 2.0–7.0 and can degrade ZEN derivatives (α-ZAL, β-ZOL, and ZAN), and the degradation rates of ZEN and ZAN are greater than those of α-ZAL and β-ZOL. The degradation rate of FSZ was 78.43% after incubation with corn flour containing 1.0 μg/mL ZEN for 28 h, which showed a good degradation effect (Ji et al., 2022).

ZenH is a novel enzyme from *Aeromicrobium* sp. HA that can degrade ZEN and exhibits the highest similarity (21.52% homology) to the lactonase ZHD607 from *Phallophora americana*. ZenH degrades ZEN into C_18_H_22_O_5_ (Figure 3I), which is an isomer of ZEN. Treatment of ZEN-contaminated corn meal with ZenH resulted in ZEN degradation rates ranging from 75.7 to 85.3% (Hu et al., 2023).

In conclusion, studies on the biodegradation and transformation mechanism of ZEN were carried out relatively later than studies on AFB_1_, so the degradation and transformation mechanism of ZEN by detoxification enzymes has not been explored. The detoxifying enzymes of ZEN are summarized in Table 2.

**Table 2:** The detoxifying enzymes of ZEN.

Enzymes	Sources	Category	Degradation mechanism	Degradation product(s)	Toxicity	References
ZHD101	*Clonostachys rosea*	Lactonase	Lactone ring hydrolysis	DHZEN	Reduce	Takahashi-Ando et al. (2002)
RmZHD	*Rhinocladiella mackenziei*					Zhou et al. (2020)
ZLHY-6	*Clonostachys rosea* 31535					Chang et al. (2020)
PR-ZHD	*Clonostachys rosea* GRZ7					Shcherbakova et al. (2020)
ZTE138	*Bacillus amyloliquefaciens* H6					Xu et al. (2020)
ZHD607	*Phialophora americana*					Yu et al. (2020)
ZENG	*Gliocladium roseum*					Zhang et al. (2020); Fang et al. (2022)
ZenA	Unknown					Gruber-Dorninger et al. (2021)
ZHD-P	*Trichoderma aggressivum*					Chen S. et al. (2021)
ZHD_LD	*Exophiala spinifera*					Gao et al. (2022)
Zhd11B	*Fonsecaea monophora*					Jiang et al. (2022)
Zhd11D	*Phialophora attinorum*					Wang Z. et al. (2023)
BVMO	*Apiotrichum mycotoxinivorans* CICC 1454	Monooxygenase	Oxidation of the ketone group	ZOM	Reduce	Mascotti et al. (2015); Sun et al. (2020)
**Enzymes**	**Sources**	**Category**	**Degradation mechanism**	**Degradation product(s)**	**Toxicity**	**References**
ZOM-HD	*Apiotrichum mycotoxinivorans* CICC 1454	Carboxylester hydrolase	Hydrolysis of the ester bond	ZOM-1	Reduce	Mascotti et al. (2015); Sun et al. (2020)
*St*DyP	*Streptomyces thermocarboxydus 41291*	Dye decolorizing peroxidase	Hydroxylation of the aromatic group	15-OH-ZEN, 13-OH-ZEN-quinone	Reduce	Qin et al. (2021b)
CotA	*Bacillus licheniformis*	Laccase	Hydroxylation of the aromatic group	15-OH-ZEN, 13-OH-ZEN-quinone	Reduce	Guo et al. (2022)
*Po*DyP4	*Pleurotus ostreatus*	Dye decolorizing peroxidase	Hydroxylation of the aromatic group	15-OH-ZEN, 14-OH-ZEN-quinone, 13,15-OH-ZEN, ZEN dimer, and 15-OH-ZEN dimer	Reduce	Ding et al. (2024)
SHP	Soybean hulls	Peroxidase	Hydroxylation of the aromatic group	13-OH-ZEN, 13-OH-ZEN-quinone	Reduce	Guo et al. (2024)
FSZ	*Aspergillus niger*	Unknown	Breaking the lactone bond	C_18_H_26_O_4_	Reduce	Ji et al. (2022)
ZenH	*Aeromicrobium* sp. HA	Unknown	Breaking the lactone bond	C_18_H_22_O_5_	Reduce	Hu et al. (2023)

### 2.3 Deoxynivalenol

**Figure 4:**
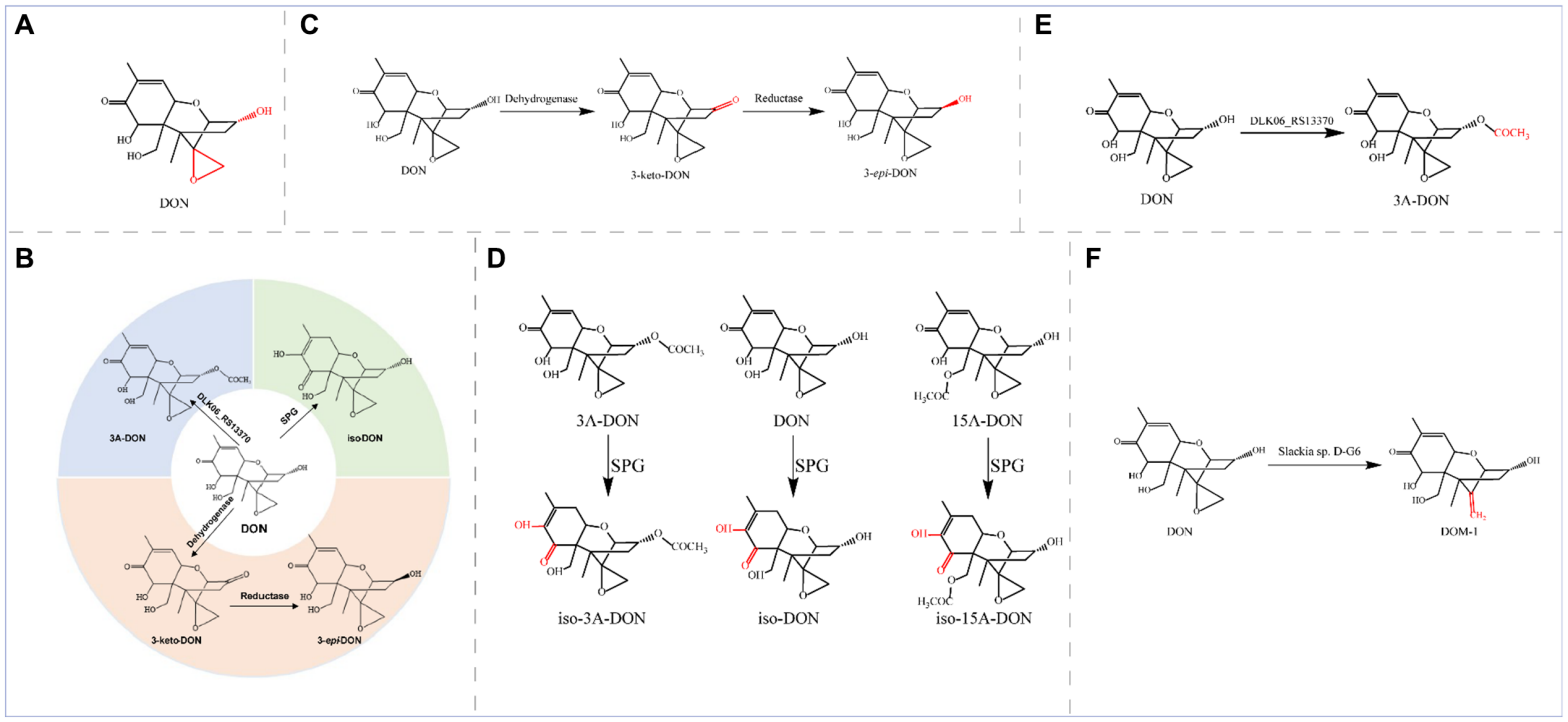
**(A)** Structural formula of DON and its corresponding detoxification enzyme action groups. The red group is the detoxifying enzyme action group. **(B)** Main degradation and transformation products of DON. **(C)** Degradation of DON by dehydrogenase and reductase. **(D)** Degradation of DON and its derivatives by SPG. **(E)** Degradation of DON by the enzyme encoded by DLK06_RS13370. **(F)** Degradation of DON by D-G6.

Deoxynivalenol (DON), also known as vomitoxin, is a class of sesquiterpenoid metabolites produced by *Fusarium* fungi and is among the typical trichothecene mycotoxins (Figure 4A).

This toxin is mainly found in grains, such as wheat, corn, and barley, that are contaminated with *Fusarium* and potentially jeopardize the health of both humans and animals (Mishra et al., 2020; Tu et al., 2023). High concentrations of deoxynivalenol can cause decreased appetite, digestive system problems, and immune system damage (Gao Y. et al., 2020; Chen et al., 2023). In severe cases, deoxynivalenol can also result in teratogenicity, carcinogenicity, and immunotoxicity (Yao and Long, 2020). Different animals exhibit different sensitivities to DON, with pigs being particularly sensitive to DON (Holanda and Kim, 2021). The main toxic groups of DON are the C12-13 epoxy ring and the OH group at C3 (Figure 4A), and the degradation and transformation of these two groups are crucial for biological detoxification. The enzymatic elimination of DON can oxidize the OH group at C3 in DON to generate the corresponding ketone group, 3-keto-DON. Alternatively, isomerization of the C3 atom can generate the epimer of DON (3-*epi*-DON) or transform a low-toxicity 3-keto-DON intermediate to 3-*epi*-DON, which is almost nontoxic; iso-DON can also be generated through isomerization. These methods can significantly reduce the physiological toxicity of DON. The main degradation and transformation products of DON are as follows (Figure 4B): dehydrogenases oxidize DON to 3-keto-DON, and then reductases isomerize 3-keto-DON to 3-*epi*-DON; glyoxalase isomerizes DON to iso-DON; and acyltransferases convert DON to 3-acetyl-deoxynivalenol (3-ADON).

#### 2.3.1 Oxidoreductase

Oxidoreductases coordinate detoxification, and dehydrogenases and reductases catalyze the conversion of DON through a two-step sequential reaction. In this reaction, the dehydrogenases first oxidize DON to 3-keto-DON and then the reductases reduce 3-keto-DON to the final product, 3-*epi*-DON, to epimerize the C3 atom (Figure 4C).

The OH group is oxidized and isomerized at C3 by two DON detoxification enzymes from *Devosia mutans* 17-2-E-8, a pyrroloquinoline quinone (PQQ)-dependent dehydrogenase (DepA) and an NADPH-dependent aldo-keto reductase (DepB) (He et al., 2016; Carere et al., 2018a). Specifically, DepA oxidizes DON to 3-keto-DON (Yang H. et al., 2022), DepB reduces 3-keto-DON to 3-*epi*-DON (Carere et al., 2018b), and the intermediate product 3-keto-DON is an order of magnitude less toxic than DON; in contrast, the final product (3-*epi*-DON) is virtually nontoxic. Two highly active DON detoxifying enzymes are also present in *Devosia* strain D6-9 and catalyze sequential reactions; specifically, the quinone-dependent DON dehydrogenase QDDH oxidizes DON to 3-keto-DON, and two NADPH-dependent aldo-keto reductases (AKR13B2 and AKR6D1) convert 3-keto-DON to 3-*epi*-DON (He et al., 2020). Deoxynivalenol dehydrogenase (DDH) from *Pelagibacterium halotolerans* ANSP101 shares 57.55% sequence identity with the dehydrogenase DepA from *Devosia,* which oxidizes DON to 3-keto-DON using phenazine methosulfate (PMS), dichlorophenolindophenol (DCPIP), or pyrroloquinoline quinone (PQQ) as the hydrogen acceptor. The TDDH mutant can degrade DON over a wide pH range (6.0–11.0) and temperature range (20–45°C) in the presence of the same hydrogen receptor and possesses a greater ability to degrade DON (Qin et al., 2022). DepB_Rleg_ from *Rhizobium leguminosarum*, a member of the new aldo-keto reductase family AKR18, is also an NADPH-dependent DON detoxification enzyme. DepB_Rleg_ converts 3-keto-DON to 3-*epi*-DON and DON in diastereomeric ratios of 67.2 and 32.8%, respectively (Abraham et al., 2022b). Sorbose dehydrogenase (SDH) from *Ketogulonicigenium vulgare* Y25 can effectively convert DON to 3-keto-DON, a substance in which DON loses four hydrogen atoms; the SDH mutants F103L and F103A were more effective, with 5- and 23-fold increases in V_max_, respectively (Li D. et al., 2023). SDH and its mutants exhibit excellent thermal stability and activity over wide pH and temperature ranges, with potential applications in detoxifying DON. *Yo*DDH, a new DON-degrading enzyme from *Youhaiella tibetensis*, showed the highest degradation activity of DON at 40°C and pH 4.5 in the presence of Ca^2+^ and PQQ, and *Yo*DDH ultimately degraded 73% of DON (100 μM) when incubated under optimum conditions for 3 h (Shi et al., 2024).

#### 2.3.2 Transferase

Transferases can catalyze the detoxification of DON by transferring specific groups. The specialized glyoxalase I from *Gossypium hirsutum* (SPG) exhibits the highest degradation activity at pH 9.0 and 55°C; in addition, SPG can reduce the toxicity of DON by isomerization to transfer the C8 carbonyl to C7 and double bonds from C9-C10 to C8-C9 to generate the less toxic iso-DON. Furthermore, SPG recognizes the DON derivatives 15A-DON and 3A-DON; generating iso-15A-DON and iso-3A-DON, respectively (Figure 4D). The SPG^Y62A^ mutant was more efficient at detoxifying DON, 3A-DON, and 15A-DON (the catalytic activity of the enzyme increased by more than 70%) (Huang et al., 2020; Hu et al., 2022).

*Acinetobacter pittii* S12, which was isolated and characterized from soil samples, also degraded DON with a high degradation rate of 78.32%. The DLK06_RS13370 gene, a pivotal gene for DON detoxification in the strain, was expressed in *E. coli*, and the resulting recombinant protein exhibited acyltransferase activity; this enzyme converted a hydroxyl group into an acetyl moiety, thereby converting DON to 3-ADON (Figure 4E) (Liu et al., 2023b).

Some strains can also de-epoxidize C12-C13, such as the novel DON detoxifying bacterium, *Slackia* sp. D-G6, isolated from chicken intestines, in which D-G6 de-epoxidizes DON into a nontoxic form called DOM-1 (Figure 4F) (Gao X. et al., 2020).

The detoxifying enzymes of DON are summarized in Table 3.

**Table 3:** The detoxifying enzymes of DON.

Enzymes	Sources	Category	Substrate	Degradation product(s)	Toxicity	References
DepA	*Devosia mutans* 17-2-E-8	Dehydrogenase	DON	3-keto-DON	Reduce	Yang H. et al. (2022)
DepB	*Devosia mutans* 17-2-E-8	Aldo-keto reductase	3-keto-DON	3-*epi*-DON	Almost no toxicity	Carere et al. (2018b)
QDDH	*Devosia mutans* D6-9	Dehydrogenase	3-keto-DON	3-keto-DON	Reduce	He et al. (2020)
AKR13B2	*Devosia mutans* D6-9	Reductase	3-keto-DON	3-*epi*-DON	Almost no toxicity	He et al. (2020)
AKR6D1	*Devosia mutans* D6-9	Reductase	3-keto-DON	3-*epi*-DON	Almost no toxicity	He et al. (2020)
DDH	*Pelagibacterium halotolerans* ANSP101	Dehydrogenase	DON	3-keto-DON	Reduce	Qin et al. (2022)
DepB_Rleg_	*Rhizobium leguminosarum*	Aldo-keto reductase	3-keto-DON	3-*epi*-DON	Almost no toxicity	Abraham et al. (2022b)
SDH	*Ketogulonicigenium vulgare* Y25	Dehydrogenase	DON	3-keto-DON	Reduce	Li Y. et al. (2023)
*Yo*DDH	*Youhaiella tibetensis*	Dehydrogenase	DON	3-keto-DON	Reduce	Shi et al. (2024)
SPG	*Gossypium hirsutum*	Glyoxalase	DON	iso-DON	Reduce	Huang et al. (2020); Hu et al. (2022)
DLK06_RS13370	*Acinetobacter pittii* S12	Acyltransferase	DON	3A-DON	Reduce	Liu et al. (2023b)

### 2.4 Ochratoxins

**Figure 5:**
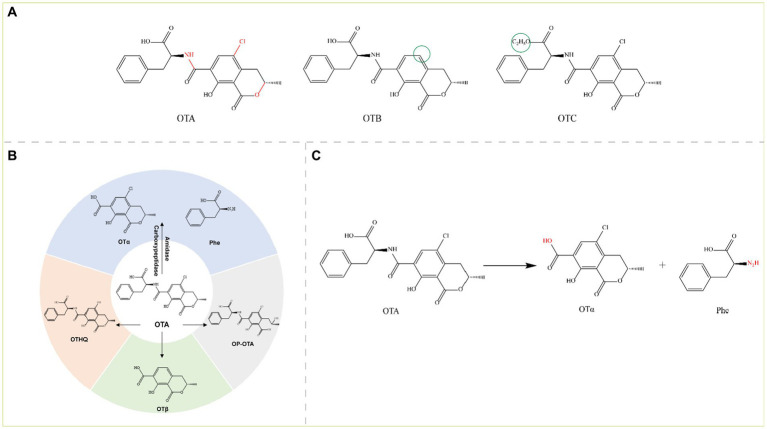
**(A)** Structural formula of OTA and its derivatives. The red group is the detoxifying enzyme action group and the green group shows the difference between OTA derivatives and them. OTA, Ochratoxin A; OTB, Ochratoxin B; and OTC, Ochratoxin C. **(B)** Main degradation transformation products of OTA. **(C)** Degradation of OTA by carboxypeptidase.

Ochratoxins are a group of isocoumarin derivative compounds produced mainly by *Aspergillus* and *Penicillium* fungi. There are three main groups of ochratoxins: ochratoxin A (OTA), ochratoxin B (OTB), and ochratoxin C (OTC) (Figure 5A). Among these toxins, OTA is the most common, ubiquitous, and virulent mycotoxin in the ochratoxin family (Mwabulili et al., 2023).

The ochratoxin skeleton consists of a dihydroisocoumarin group linked to phenylalanine by an amide bond, and OTA and OTC also contain a p-chlorophenol structure (Freire et al., 2020). OTA is commonly found in grains (wheat, maize, barley, oats, rye, rice, etc.) contaminated by *Aspergillus* and *Penicillium*. OTA is a potent nephrotoxin and is also hepatotoxic, carcinogenic, teratogenic, genotoxic, immunotoxic, and embryotoxic (Kumar et al., 2020; Gu et al., 2021). The main toxic groups of OTA are amide bonds, isocoumarin rings, lactone rings, and chlorine atoms, and the toxins are detoxified by several reactions, including hydrolysis of amide bonds, hydroxylation, ring opening of the lactone ring, and dichlorination. These reactions are accomplished mainly with enzymes such as amidases, peptidases (aminopeptidases and carboxypeptidases) and lipases. Currently, the production of ochratoxin α (OTα) and L-β-phenylalanine (Phe) by the hydrolysis of amide bonds is the most important mechanism for the degradation and detoxification of OTA. However, other pathways can lead to the detoxification of OTA such as dechlorination to OTB and further degradation to ochratoxin β (OTβ), hydroxylation of the isocoumarin ring to ochratoxin hydroquinone (OTHQ), and lactone ring-opening to lactone-opened OTA (OP-OTA) (OP-OTA is more toxic than OTA) (Figure 5B) (Wu et al., 2011).

#### 2.4.1 Carboxypeptidases

Carboxypeptidase detoxifies the amide bond of OTA, which hydrolyzes OTA to the virtually nontoxic OTα and the nontoxic Phe (Figure 5C); OTα is almost a thousand times less toxic than OTA. Cleavage (hydrolysis) of OTA amide bonds by carboxypeptidases is the main pathway for OTA detoxification.

Pitout (1969) discovered that carboxypeptidase A (CPA) from bovine pancreas, which was the first enzyme found to degrade OTA, could degrade OTA. In a study on CPA in contaminated wheat flour, researchers showed that CPA from three different sources (animal, vegetable, and microbial) could degrade OTA, and the best results were observed with microbial sources (Kupski et al., 2018). However, the poor thermal stability of native CPA limits its application; therefore, to produce efficient catalytic CPAs with *in vitro* stability, researchers have mostly genetically engineered the CPA gene and performed heterologous expression. Compared with those of other OTA degradation enzymes, the catalytic mechanism and crystal structure of CPA are well established. Lu Xiong constructed a mature CPA (M-CPA) without a propeptide or signal peptide and expressed it in *P. pastoris*. M-CPA degraded OTA up to 93.36%, with an optimum pH of 8 and temperature of 40°C. M-CPA could effectively degrade OTA in red wine (Xiong et al., 2020). CPA can be used to detoxify OTA in contaminated foods and feeds, and it shows good potential for various applications. Carboxypeptidase Y (CPY), produced by *S. cerevisiae*, is an analogous enzyme to CPA and breaks down OTA to the less toxic OTα; however, the native CPY is also defective, with very low specific activity and a very slow OTA hydrolysis reaction (only 52% of the OTA was converted to OTα in 5 days) (Abrunhosa et al., 2010). In recent years, researchers have identified several other carboxypeptidases, such as the carboxypeptidase cp4 gene from *Lysobacter* sp. CW239, which is expressed in *E. coli* and can degrade OTA to produce OTα; however, the OTA degradation rate of rCP4 after 24 h was only 36.8% (Wei W. et al., 2020). Moreover, the carboxypeptidase DacA gene from *Bacillus subtilis* CW14 was expressed in *E. coli*, and recombinant DacA converted OTA to OTα with a degradation rate of 71.3% within 24 h at 30°C and pH 7.0 (Xu et al., 2021). The carboxypeptidase BnOTase2 from the *Brevundimonas naejangsanensis* ML17 strain could hydrolyze OTA to OTα and OTB to OTβ with a degradation rate of 100% (Peng et al., 2022, 2023). In addition, *Af*OTH from *Alcaligenes faecalis* subsp. *phenolicus* DSM 16503^T^ exhibited both amidase and carboxypeptidase dual activity, with higher activity at pH 6.0–9.0 and 30–60°C (Sánchez-Arroyo et al., 2024).

#### 2.4.2 Amidohydrolases

Amidohydrolases are also a typical class of OTA-degrading enzymes that produce OTα by hydrolyzing amide bonds. The novel microbial ochratoxin amidase gene from *Aspergillus niger* was cloned and homologously expressed in *Aspergillus niger*, and the recombinant protein ochratoxinase (OTase) showed optimum activity at pH 6.0 and 66°C; compared with carboxypeptidase A and Y, OTase hydrolyzed OTA more efficiently (Dobritzsch et al., 2014). The novel degrading enzyme N-acyl-L-amino acid amidohydrolase (*Af*OTase) from *A. faecalis* DSM 16503 belongs to the peptidase family M20, and recombinant *Af*OTase (r*Af*OTase) efficiently degrades OTA to OTα at an optimum temperature and pH of 50°C and 6.5, respectively (Zhang et al., 2019). The amidohydrolase ADH3 from *Stenotrophomonas acidaminiphila* is the most potent OTA detoxification enzyme reported to date; ADH3 hydrolyzes OTA to OTα and Phe, and recombinant ADH3 (1.2 μg/mL) expressed in *E. coli* completely degrades 50 μg/L OTA in 90 s. However, several hours are needed for other OTA-degrading enzymes to fully degrade OTA (Luo et al., 2022). The catalytic activity of the rationally modified S88E mutant expressed in *P. pastoris* increased by 3.7-fold (Dai et al., 2023). The salicylate 1,2-dioxygenase from *Pseudaminobacter salicylatoxidans* DSM 6986^T^ (*Ps*SDO) is a versatile metalloenzyme with both dioxygenase and amide hydrolase catalytic activities; through its amidase activity, this enzyme can hydrolyze the amide bond of OTA to produce OTα and L-β-phenylalanine (Sánchez-Arroyo et al., 2023). Chr1-3858681-3267, a metallo-dependent amidohydrolase from the aerobic gram-negative bacterium *Stenotrophomonas* sp. 043-1a, also degrades OTA (Gonaus et al., 2023).

#### 2.4.3 Other hydrolyzing enzymes

Lipases and Nudix family hydrolases can also detoxify OTA. The Nudix hydrolase Nh-9 gene from the *Bacillus velezensis* IS-6 strain was expressed in *E. coli*, the recombinant Nh-9 enzyme was purified to degrade 1.0 μg/mL OTA by 68% within 24 h at 37°C and pH 7.0, and OTA was detoxified to OTα; Fe^2+^ and Cu^2+^ enhanced the degradation of OTA (Jahan et al., 2023). Amano lipase A from *Aspergillus niger* (ANL) and porcine pancreas lipase (PPL) hydrolyzed OTA, but the enzymes exhibited different degrees of hydrolysis; ANL completely degraded OTA and OTB after 3 and 10 h, respectively, whereas PPL failed to completely degrade OTA (only 43% of OTA was degraded after 25 h) but completely degraded OTB within 9 h (Santos et al., 2023).

Among the above OTA degradation and transformation pathways, the degradation pathway with OTα as the end product is recognized as the most effective degradation and detoxification pathway. The reported detoxification enzymes for OTA are summarized in Table 4.

**Table 4:** The detoxifying enzymes of OTA.

Enzymes	Sources	Category	Degradation product(s)	Toxicity	References
CPA	Bovine pancreas	Carboxypeptidase	OTα	Almost no toxicity	Pitout (1969); Kupski et al. (2018)
M-CPA	Bovine pancreas	Carboxypeptidase			Xiong et al. (2020)
CPY	*Saccharomyces cerevisiae*	Carboxypeptidase			Abrunhosa et al. (2010)
cp4	*Lysobacter* sp. CW239	Carboxypeptidase			Wei W. et al. (2020)
DacA	*Bacillus subtilis* CW14	Carboxypeptidase			Xu et al. (2021)
BnOTase1-3	*Brevundimonas naejangsanensis* ML17	Peptidase			Peng et al. (2022, 2023)
BnOTase4	*Brevundimonas naejangsanensis* ML17	Amidase			Peng et al. (2022, 2023)
*Af*OTH	*Alcaligenes faecalis* subsp. *phenolicus* DSM 16503^T^	Carboxypeptidase			Sánchez-Arroyo et al. (2024)
*Af*OTase	*Alcaligenes faecalis DSM 16503*	Amidohydrolase			Zhang et al. (2019)
ADH3	*Stenotrophomonas acidaminiphila*	Amidase			Luo et al. (2022); Dai et al. (2023)
*Ps*SDO	*Pseudaminobacter salicylatoxidans* DSM 6986^T^	Amidase			Sánchez-Arroyo et al. (2023)
Chr1-3858681-3267	*Stenotrophomonas* sp. 043-1a	Amidase			Gonaus et al. (2023)
Chr1-3858681-771	*Stenotrophomonas* sp. 043-1a	Aminopeptidase			Gonaus et al. (2023)
Nh-9	*Bacillus velezensis* IS-6	Nudix hydrolase			Jahan et al. (2023)
ANL	*Aspergillus niger*	Lipase			Santos et al. (2023)

### 2.5 Patulin

**Figure 6:**
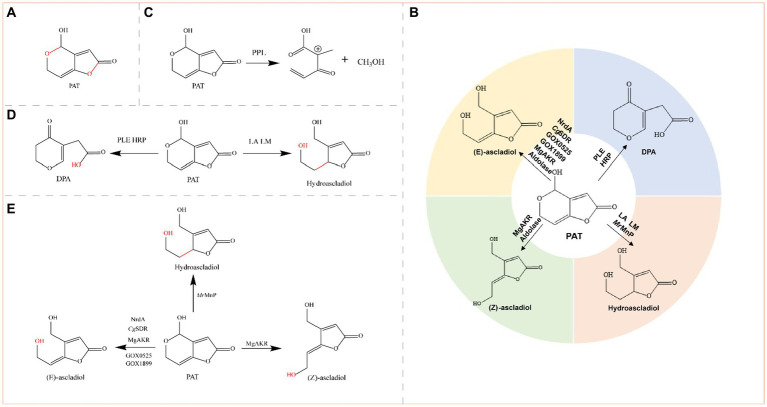
**(A)** Structural formula of PAT (patulin) and its corresponding detoxification enzyme action groups. The red group is the detoxifying enzyme action group. **(B)** Main degradation transformation products of PAT. **(C)** Degradation of PAT by PPL. **(D)** Possible degradation of PAT by PLE, HRP, LA, and LM. **(E)** Degradation of PAT by oxidoreductases.

Patulin (PAT) is a toxic polyketide lactone compound produced primarily by *Aspergillus*, *Penicillium*, and *Byssochlamys* fungi (Figure 6A) (Mahato et al., 2021).

Patulin is produced primarily in rotten fruits such as apples, pears, blueberries, cherries, peaches, plums, strawberries, and mulberries. Most human intake of PAT occurs via apples and apple products because rotten apples contain relatively high concentrations of PAT (Zheng et al., 2021). Animal experiments have shown that PAT affects a variety of organs, including the gut, liver, and kidneys (Wei C. et al., 2020). The main toxic groups of PAT are hemiacetal and lactone rings (Figure 6A). PAT can be reduced to E-ascladiol by short-chain dehydrogenases or the aldo-keto reductase family of enzymes, which can be converted to Z-ascladiol catalyzed by cellular sulfhydryl compounds, both of which are virtually nontoxic (Sekiguchi et al., 1983). E-ascladiol can also be reduced by manganese peroxidase to hydroascladiol, a much less toxic compound. Degradation of PAT to E-ascladiol appears to be more prevalent, but certain esterases and peroxidases convert PAT to deoxypatulinic acid (DPA), which is significantly less toxic than PAT (Tannous et al., 2017). Overall, the detoxification of PAT is achieved by the conversion of PAT to less toxic degradation products such as DPA, (E)-ascladiol, (Z)-ascladiol, and hydroascldiol by lipases, transferases, oxidoreductases, and aldolases (Figure 6B).

#### 2.5.1 Lipase

Lipase detoxifies PAT by destroying its lactone and hemiacetal rings. PPL was used to degrade PAT in pear juice at a dose of 0.02 g/mL, and was effective at 40°C within 24 h; the maximum degradation rate was 61.38% and no decrease in the quality or nutrition of the pear juice was observed (Figure 6C) (Xiao et al., 2019). The efficiency of the enzyme is greatly increased at lower PAT concentrations, so it is important to control PAT contamination before it spreads. The efficiency of the enzyme can also be improved if an immobilized PPL enzyme is applied (Yan et al., 2024).

The lipase RL12 from *Ralstonia* sp. strain SL312, 100 μg/mL RL12, degraded more than 80% of the PAT in apple juice within 24 h at an optimum pH of 7.5 and a temperature of 37°C. In addition, the nutritional and sensory qualities of apple juice are improved by the enzymatic processes (He et al., 2022).

Commercial enzymes are now available for the degradation of PAT in fruit juices. For example, esterase from porcine liver (PLE), peroxidase from horseradish (HRP), lipase A from *Aspergillus niger* (LA) and lipase M from *Mucor javanicus* (LM) degrade 97.8, 53.2, 76.3, and 68.2% of PAT in apple juice, respectively, and the effects of the four enzymes on the quality of apple juice are essentially within acceptable limits. Among these enzymes, PLE and HRP might degrade PAT by breaking its lactone ring, generating DPA as a degradation product; LA and LM might degrade PAT by destroying its hemiacetal structure, generating hydroascladiol as the degradation product (Figure 6D) (Liu X. et al., 2023).

#### 2.5.2 Transferase

The main transferase enzymes that exert detoxifying effects on PAT are phosphoribosyltransferase and methyltransferase, but their detoxification mechanisms and degradation products are unknown and should be further explored. The orotate phosphoribosyltransferase (OPRTase) from *Rhodotorula mucilaginosa* was reported to effectively degrade PAT in apple juice at 25°C. OPRTase (0.15 g/L) degraded 1 mg/L PAT, and the final PAT degradation rate was greater than 80% (1 mg/L) (Tang et al., 2019). *Pc*CRG1 from *Pichia caribbica*, an S-adenosylmethionine-dependent methyltransferase, degraded PAT from 20 μg/mL to undetectable levels within 72 h. The *Pc*CRG1 enzyme was also found to effectively degrade PAT in apple juice (Wang K. et al., 2019).

#### 2.5.3 Oxidoreductase

Oxidoreductases degrade PAT to either hydroascladiol or ascladiol (E-ascladiol or Z-ascladiol); manganese peroxidases degrade PAT to hydroascladiol and ribonucleoside diphosphate reductases, short-chain dehydrogenase/reductases (SDRs), and aldo-keto reductases degrade PAT to ascladiol (Figure 6E).

Ribonucleoside diphosphate reductase (NrdA) from *Enterobacter cloacae* subsp. TT-09 converts PAT to E-ascladiol (Xing et al., 2020). The short-chain dehydrogenase/reductase (SDR) gene *Cg*SDR, which was cloned from *Candida guilliermondii*, was heterologously expressed in *E. coli*. The resulting recombinant *Cg*SDR (150 μg/mL) with NADPH dependence converted 80% of the PAT in apple juice to E-ascladiol, and the biodegradation process did not affect the quality of the apple juice (Xing et al., 2021). After *Cg*SDR was covalently linked to dopamine/polyethyleneimine-codeposited magnetic Fe_3_O_4_ particles for immobilization, the thermal and storage stabilities of the enzyme, proteolysis resistance, and reusability were greatly improved (Xing et al., 2023). The NADPH-dependent short-chain dehydrogenases GOX0525 and GOX1899 from *Gluconobacter oxydans* ATCC 621, both of which belong to the short-chain dehydrogenase/reductase (SDR) family, completely converted PAT to E-ascladiol within 24 h (Chan et al., 2022). *Mr*MnP from *Moniliophthora roreri* was the first manganese peroxidase found to degrade PAT efficiently; the *Mr*MnP gene was heterologously expressed in *P. pastoris*, and *Mr*MnP produced the most rapid degradation of PAT in the malonate/Mn^2+^ system. At 0.5 U/mL, *Mr*MnP completely removed 5 mg/L PAT within 5 h and eliminated up to 95% of the PAT in apple juice after 24 h (Wang S. et al., 2022). The novel NADPH-dependent PAT aldo-keto reductase *Mg*AKR from *Meyerozyma guilliermondii* was expressed in *E. coli*, and *Mg*AKR converted PAT to ascladiol. At an optimum reaction temperature of 16°C and pH 6.0, 88% of the PAT (5 μg/mL) in fresh pear juice was degraded by *Mg*AKR (300 μg/mL) within 4 h, and the biodegradation process did not affect the quality of the fresh pear juice (Zhang et al., 2024).

#### 2.5.4 Aldolase

Aldolase degrades PAT to form ascladiol, and aldolase from *R. mucilaginosa* degrades 2 mg/L PAT in apple juice at 0.7 mg/mL. The optimum pH and temperature were 7.0 and 25°C, respectively, but the enzyme was stable within the pH range of 5.5–7.0 and from 4 to 25°C. In addition, there was no significant difference in the quality parameters or volatile components of apple juice before and after degradation, and the purified aldolase cloned and recombinantly expressed in *E. coli* degraded more than 99% of the PAT (Yang et al., 2021; Li et al., 2022b).

Compared with its degradation by transferases, the degradation of PAT by lipases, oxidoreductases and aldolases has been more thoroughly studied; however, the degradation mechanism and products of PAT by transferases are unclear and should be further explored. Some of the above enzymes synergized product processing with PAT degradation and did not reduce the product quality during the mycotoxin degradation process, leading to an ideal state. The enzymes that detoxify PAT are summarized in Table 5.

**Table 5:** The detoxifying enzymes of PAT.

Enzymes	Sources	Category	Degradation product(s)	Toxicity	References
PPL	Porcine pancreatic	Lipase	C_7_H_11_O_4_^+^	Reduce	Xiao et al. (2019)
RL12	*Ralstonia* sp. SL312	Lipase	C_7_H_11_O_4_^+^	Reduce	He et al. (2022)
PLE	*Porcine liver*	Esterase	DPA	Reduce	Liu X. et al. (2023)
HRP	Horseradish	Peroxidase	DPA	Reduce	Liu X. et al. (2023)
LA	*Aspergillus niger*	Lipase	hydroascladiol	Reduce	Liu X. et al. (2023)
LM	*Mucor javanicus*	Lipase	hydroascladiol	Reduce	Liu X. et al. (2023)
OPRTase	*Rhodotorula mucilaginosa*	Phosphoribosyltransferase	Unknown	Unknown	Tang et al. (2019)
*Pc*CRG1	*Pichia caribbica*	Methyltransferase	Unknown	Unknown	Wang K. et al. (2019)
NrdA	*Enterobacter cloacae sub*sp. TT-09	Ribonucleoside diphosphate reductase	E-ascladiol	Reduce	Xing et al. (2020)
*Cg*SDR	*Candida guilliermondii*	Short-chain dehydrogenase/reductase	E-ascladiol	Reduce	Xing et al. (2021)
GOX0525	*Gluconobacter oxydans* ATCC 621	Short-chain dehydrogenase	E-ascladiol	Reduce	Chan et al. (2022)
GOX1899	*Gluconobacter oxydans* ATCC 621	Short-chain dehydrogenase	E-ascladiol	Reduce	Chan et al. (2022)
*Mr*MnP	*Moniliophthora roreri*	Manganese peroxidase	hydroascladiol	Reduce	Wang S. et al. (2022)
MgAKR	*Meyerozyma guilliermondii*	Aldo-keto reductase	Ascladiol	Reduce	Zhang et al. (2024)
Aldolase	*Rhodotorula mucilaginosa*	Aldolase	Ascladiol	Reduce	Yang et al. (2021); Li N. et al. (2022)

### 2.6 Fumonisins

**Figure 7:**
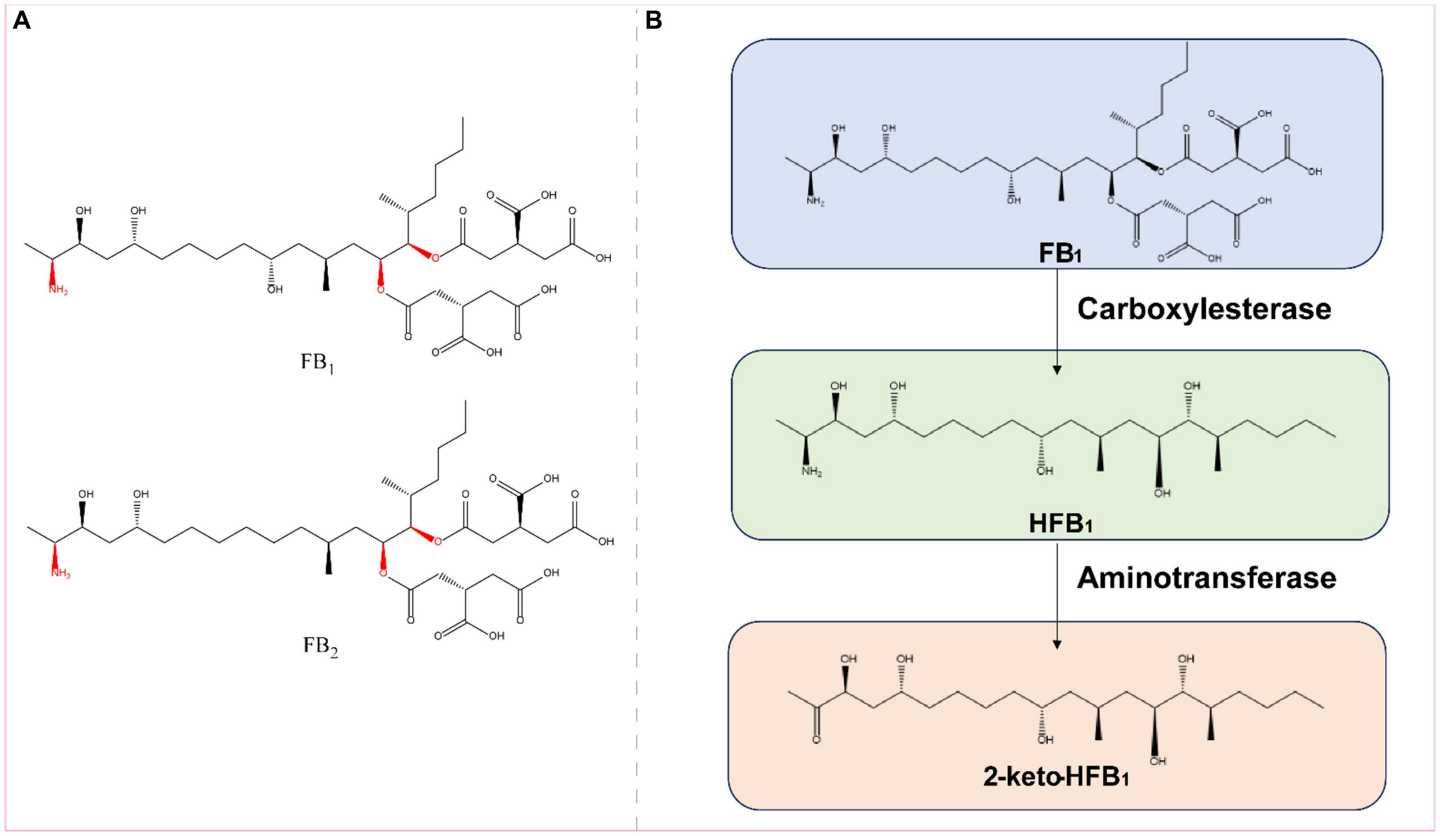
**(A)** Structural formula of FB_1_ (fumonisin B_1_) and its corresponding detoxification enzyme action groups. The red group is the detoxifying enzyme action group. **(B)** Main degradation transformation products of FB_1_.

Fumonisin is a water-soluble metabolite produced by *Fusarium* fungi, such as *Fusarium verticillioides* and *Fusarium proliferatum* (Rudyk et al., 2023). The fumonisins include fumonisin B_1_ (FB_1_), fumonisin B_2_ (FB_2_), fumonisin B_3_ (FB_3_), fumonisin B_4_ (FB_4_), fumonisin A_1_ (FA_1_), and fumonisin A_2_ (FA_2_), of which FB_1_ and FB_2_ are more toxic (Figure 7A); in contrast, several other toxins have very low concentrations of contaminants and are less toxic (Ben Taheur et al., 2019). FB_1_ is the most abundant and toxic variant of naturally contaminated maize, accounting for 70–80% of the total fumonisins. Recent studies have focused on the detoxification of FB_1_; other toxins are less frequently reported.

Fumonisin B_1_ is found primarily on grains such as corn and rice that are contaminated with *Fusarium*. Exposure to FB_1_ can cause varying degrees of damage to the nervous, respiratory, digestive, and reproductive systems (Gao et al., 2023). FB_1_ is toxic to the liver, kidneys, and mammalian cells (Chen J. et al., 2021). The main toxic groups of FB_1_ are the C14- and C15-tricarboxylic acid groups and the C2-amino group (Figure 7A). FB_1_ can be detoxified by the synergistic action of carboxylesterases and aminotransferases, and this detoxification process is divided into two steps: in the first step, the C-14 and C-15 tricarboxylic acid groups are removed in the presence of carboxylesterases, converting FB_1_ to hydrolyzed FB_1_ (HFB_1_); in the second step, HFB_1_ is converted to 2-keto-HFB_1_ in the presence of aminotransferases (Figure 7B) (Blackwell et al., 1999; Heinl et al., 2010, 2011).

The carboxylesterases involved in FB_1_ detoxification include the fumonisin esterases FumD and FumDSB. Among these enzymes, FumD is a commercial enzyme used in the animal feed industry that effectively reduces FB_1_ in maize and maize products, and the detoxification of FB_1_ by FumD is specific and irreversible (Alberts et al., 2019, 2021). A novel carboxylesterase from the bacterium *Sphingomonadales* FumDSB was expressed in *E. coli*, and under ideal conditions, FumDSB degraded FB_1_ up to 100%. In addition, FumDSB showed high degradation activity, excellent pH stability, good thermal stability at 30–40°C and activity over a wide pH range (6.0–9.0); thus, FumDSB is well suited for use under physiological conditions in animals and can be developed as a promising food and feed additive. FumDSB can alleviate the inflammatory response induced by FB_1_ in growing pigs when it is added to the feed (Li et al., 2021; Liu et al., 2022).

After FB_1_ is hydrolyzed to HFB_1_ by carboxylesterases, it combines with aminotransferases and continues to remove C2-amino acids. The aminotransferase FumIS from *Sphingopyxis* sp. MTA144 and the aminotransferase FumIB from Bacterium ATCC 55552 efficiently removed C2-amino acids from HFB_1_. Feed enzymes can be used to degrade naturally antinutritive substances in agricultural products or to improve the nutritional value of feed. FumI may be suitable for future use as a feed enzyme (Hartinger et al., 2010, 2011). Yue Wang reported three fumonisin detoxifying aminotransferases, FumTSTA, FumUPTA, and FumPHTA, that share 61–74% sequence identity with FumIS and FumIB and exhibit good pH and thermal stabilities (Wang Y. et al., 2023).

Because the detoxification of FB_1_ requires the sequential action of carboxylesterases and aminotransferases, Kailin Li generated the fusion enzyme FUMDI by connecting the carboxylesterase gene (fumD) with the aminotransferase gene (fumI) through PCR. Researchers subsequently expressed the enzyme in *P. pastoris*, in which it almost completely degraded 5 μg/mL FB_1_ in 24 h. The experiments demonstrated that the fusion enzyme FUMDI and its degradation products were not toxic to human gastric epithelial cells (Li K. et al., 2022). The detoxification enzymes of FB_1_ are summarized in Table 6.

**Table 6:** The detoxifying enzymes of FB_1_.

Enzymes	Sources	Category	Substrate	Degradation product(s)	Toxicity	References
FumD	Unknown	Carboxylesterase	FB_1_	HFB_1_	Reduce	Alberts et al. (2019, 2021)
FumDSB	*Sphingomonadales*	Carboxylesterase	FB_1_	HFB_1_	Reduce	Li et al. (2021); Liu et al. (2022)
FumIS	*Sphingopyxis* sp. MTA144	Aminotransferase	HFB_1_	2-keto-HFB_1_	Reduce	Hartinger et al. (2010, 2011)
FumIB	Bacterium ATCC 55552	Aminotransferase	HFB_1_	2-keto-HFB_1_	Reduce	Hartinger et al. (2010, 2011)
FumTSTA	Unknown	Aminotransferase	HFB_1_	2-keto-HFB_1_	Reduce	Wang Y. et al. (2023)
FumUPTA	Unknown	Aminotransferase	HFB_1_	2-keto-HFB_1_	Reduce	Wang Y. et al. (2023)
FumPHTA	Unknown	Aminotransferase	HFB_1_	2-keto-HFB_1_	Reduce	Wang Y. et al. (2023)
FumDI	Unknown	Fusion enzyme	FB_1_	2-keto-HFB_1_	Reduce	Li K. et al. (2022)

### 2.7 Other rare toxins

There are also several mycotoxins, such as sterigmatocystin, gliotoxin, and citrinin, which are difficult to analyze and are less common in nature; therefore, enzymatic degradation by these mycotoxins is less studied and reported at present.

**Figure 8:**
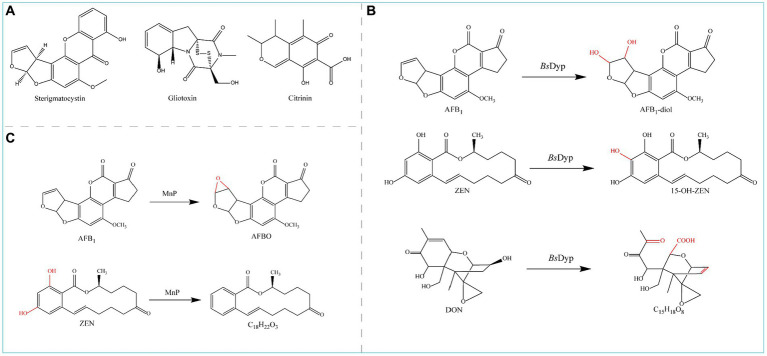
**(A)** Chemical structural formula of Sterigmatocystin, Gliotoxin, and Citrinin. **(B)** Degradation of AFB_1_ and ZEN by MnP. **(C)** Degradation of AFB_1_, ZEN, and DON by *Bs*DyP.

Sterigmatocystin (STE) (Figure 8A), a polyketide mycotoxin produced mainly by fungi (such as *Aspergillus versicolor* and *Aspergillus nidulans*), is a carcinogenic, teratogenic, and mutagenic toxin that contaminates foods such as maize, peanuts, rice, and wheat. In *Aspergillus* strains, STE is a direct precursor (O-methylsterigmatocystin) of AFB_1_ and AFG_1_ and can be rapidly converted to both substances (Zingales et al., 2020).

Gliotoxin (Figure 8A), a mycotoxin produced by *Aspergillus fumigatus*, can cause immunosuppression, genotoxicity, cytotoxicity, and apoptosis (Pena et al., 2015). Gliotoxin is synthesized by the gliotoxin oxidase GliT and can therefore be reverse degraded via its synthetic pathway (Dolan et al., 2017).

Citrinin (CTN) (Figure 8A) is a nephrotoxic compound often produced by *Penicillium*, *Aspergillus*, and *Monascus*-contaminated seeds and foodstuffs (Flajs and Peraica, 2009; Wu et al., 2022). After *R. borbori* PS45 and *E. cloacae* PS21 were cultured on mineral media supplemented with 5 ppm citrinin, minimal citrinin degradation residues were observed (Kanpiengjai et al., 2016).

## 3 Multiple strategies for degrading mycotoxins

The research examples listed in part 2 (common mycotoxins and corresponding enzymes used for detoxification) are mostly based on the degradation of one mycotoxin by one enzyme. However, foods and feeds often contain a variety of mycotoxins, and multiple mycotoxins may have synergistic and superimposed toxicological effects on humans and animals. An ideal and economical detoxification pathway to address multiple mycotoxin contaminants is the application of one or several enzymes to degrade multiple mycotoxins; in addition, multiple detoxification enzymes can be simultaneously expressed by the same host bacterium to enhance the effect of detoxification and improve the efficiency of detoxification.

### 3.1 Detoxification of multiple mycotoxins by a single enzyme

Manganese peroxidase (MnP) can simultaneously degrade four major mycotoxins, AFB_1_, ZEN, DON, and FB_1_, in the presence of a dicarboxylic acid malonate; MnP converts AFB_1_ to AFBO and ZEN to C_18_H_22_O_4_ (Figure 8B) and detoxifies DON and FB_1_, although the degradation products are unknown (Wang et al., 2019b).

The commercial peroxidase (POD) enzyme (*Armoracia rusticana*) degraded both OTA and ZEN in a model solution and beer with respective degradation rates of 27.0 and 64.9% for OTA and ZEN in the model solution and 4.8 and 10.9% in the beer after 360 min (De Oliveira Garcia et al., 2020).

The dye-decolorizing peroxidase-encoding gene *Bs*DyP from *Bacillus subtilis* SCK6 was expressed in *E. coli*, and the purified recombinant *Bs*DyP effectively degraded different types of mycotoxins such as AFB_1_, ZEN, and DON in the presence of Mn^2+^; the degradation products were AFB_1_-diol, 15-OH-ZEN, and C_15_H_18_O_8_ (Figure 8C), respectively, leading to significantly reduced toxicity (Qin et al., 2021a).

The CotA laccase gene from *Bacillus licheniformis* ZOM-1 was expressed in *E. coli*. CotA can degrade Alternaria toxin alternariol (AOH) in addition to the oxidative degradation of ZEN and AFB_1_. The optimum temperature of the CotA laccase was 80°C, and the pH was approximately 9.0 (Sun F. et al., 2023).

Lac-W, a laccase from *Weizmannia coagulans* 36D1, directly degrades AFB_1_, ZEN, DON, T-2 toxin, FB_1_, and OTA in the absence of redox mediators, and the degradation effect decreases in the following order: AFB_1_ > ZEN > DON > T-2 toxin > FB_1_ > OTA. The optimum conditions for AFB_1_ degradation by Lac-W are 30°C at pH 9.0, and the degradation products are not toxic to intestinal porcine epithelial cells (Hao et al., 2023).

The lactonase gene AttM, which can degrade AFB_1_, was expressed in *E. coli*; in addition to degrading AFB_1_, AttM also degraded OTA and ZEN at pH 8.5 and 80°C (Cheng et al., 2023).

Some enzymes can degrade both AFB_1_ and ZEN mycotoxins. *Bs*CotA laccase degraded AFB_1_ by 98.0% and ZEN by 100.0% in the presence of the mediator methyl syringate (Wang et al., 2019a). Lac-2 laccase from the fungus *Pleurotus pulmonarius* was expressed in *P. pastoris*, and recombinant Lac-2 efficiently degraded ZEN at pH 4.0–8.0 and 40–60°C. Both Lac2-ABTS and Lac2-AS were effective at degrading ZEN, and Lac2-AS was effective at degrading AFB_1_ at pH 7.0 and 37°C (99.82% degradation after 1 h) (Song et al., 2021). The *St*MCO gene was cloned form *Streptomyces thermocarboxydus* and expressed in *E. coli*, and the recombinant laccase *St*MCO directly degraded AFB_1_ and ZEN to AFQ_1_ and 13-OH-ZEN-quinone, respectively, which can be significantly accelerated in the presence of the mediator ABTS (2,2′-azino-bis(3-ethylbenzothiazoline-6-sulfonate)) (Qin et al., 2021c). The genes from *Acinetobacter nosocomialis* Y1 Porin and Peroxiredoxin degraded AFB_1_ and ZEN, producing AFD_1_ and α/β-ZAL as the degradation products, and the recombinant proteins exhibited a high degradation rate of both toxins (Adegoke et al., 2023).

The toxicity of mycotoxins is usually attributed to specific functional groups in their chemical structures; AFB_1_ and OTA both consist of a coumarin moiety, while the primary structures of AFB_1_, ZEN, OTA, and PAT are based on lactone rings. Some toxins contain the same toxic group, and certain degrading enzymes have strong group specificity but broader substrate specificity; thus, these enzymes can degrade a variety of mycotoxins with specific toxic groups. Molecular docking simulations have shown that enzymes with broad substrate specificity have substrate-binding pockets large enough to allow substrates of different structures to enter the active site of the enzyme, thereby catalyzing the degradative transformation of multiple functional groups of different mycotoxins. Therefore, the future development of enzymes that can catalyze multiple mycotoxins by expanding the substrate specificity of enzymes through genetic engineering will provide ideas for future mycotoxin detoxification.

### 3.2 Application of recombinant fusion enzyme technology

In addition, studies on the detoxification of various mycotoxins by recombinant fusion enzyme technology have increased; in this technology, two or more single genes are combined by PCR. For example, zearalenone hydrolase (ZHD) and carboxypeptidase (CP) combine to form zearalenone hydrolase-carboxypeptidase (ZHDCP) for the detoxification of OTA and ZEN. The ZHDCP enzyme completely detoxified OTA (100%) within 30 min at pH 7.0 and 30°C, degraded ZEN by 60% in 48 min at pH 7.0 and 35°C and completely detoxified ZEN in 2 h (Azam et al., 2019). Another recombinant fusion enzyme for mycotoxin detoxification was ZPF1, which was formed by linking zearalenone hydrolase and manganese peroxidase using the linker peptide GGGGS; ZPF1 was successfully secreted and expressed in a newly constructed food-grade recombinant strain of *K. lactis* GG799 (pKLAC1-ZPF1) and degraded AFB_1_ and ZEN at high rates. Thus, this enzyme has potential for application in the food industry (Xia et al., 2021).

For the expression of fusion enzymes, it is important to select host bacteria that are not harmful to humans. *E. coli*, a type of opportunistic pathogen, is sometimes unsuitable for the expression of fungal enzymes, especially those used in the food industry. In contrast, we can choose food-safe yeast as a host bacterium, which is a suitable organism for the production of food enzymes.

These enzymes do not follow the conventional approach in which one enzyme degrades one specific mycotoxin. The enzymes that can simultaneously degrade multiple mycotoxins are summarized in Table 7.

**Table 7:** Enzymes that degrade multiple mycotoxins simultaneously.

Enzymes	Sources	Category	Biodegradable mycotoxins	References
MnP	*Irpex lacteus CD2*	Manganese peroxidase	AFB_1_, ZEN, DON, and FB_1_	Wang et al. (2019b)
	*Phanerochaete*, *Chrysosporium*			
	*Ceriporiopsis subvermispora,*			
	*Nematoloma frowardii*			
POD	*Armoracia rusticana*	Peroxidase	OTA, ZEN	De Oliveira Garcia et al. (2020)
*Bs*DyP	*Bacillus subtilis* SCK6	Dye decolorizing peroxidase	AFB_1_, ZEN, and DON	Qin et al. (2021a)
CotA	*Bacillus licheniformis*	Laccase	AFB_1_, ZEN, and AOH	Sun F. et al. (2023)
Lac-W	*Weizmannia coagulans* 36D1	Laccase	AFB_1_, ZEN, DON, T-2, FB_1_, and OTA	Hao et al. (2023)
AttM	*Bacillus megaterium* HNGD-A6	Lactonase	AFB_1_, ZEN, OTA	Cheng et al. (2023)
*Bs*CotA	*Bacillus subtilis*	Laccase	AFB_1_, ZEN	Wang et al. (2019a)
Lac-2	*Pleurotus pulmonarius*	Laccase	AFB_1_, ZEN	Song et al. (2021)
*St*MCO	*Streptomyces thermocarboxydus*	Laccase	AFB_1_, ZEN	Qin et al. (2021c)
Porin	*Acinetobacter nosocomialis* Y1	Protease	AFB_1_, ZEN	Adegoke et al. (2023)
Peroxredoxin		Peroxidase	AFB_1_, ZEN	Adegoke et al. (2023)

### 3.3 Microbiological detoxification of mycotoxins

Microbiological detoxification of mycotoxins relies mainly on fungi and bacteria, which will be briefly described using the examples of *S. cerevisiae* and lactic acid bacteria (LAB). *S. cerevisiae* grows rapidly and is suitable for use in agricultural production. LAB has immunomodulatory properties that prevent the germination of mold spores, and can be used as a biological food preservative (Shetty and Jespersen, 2006). The microbiological degradation mechanism is mainly by enzymatic and degradative sorption (Abdi et al., 2021). Some strains of *S. cerevisiae* from West African fermented foods bind AFB_1_ in significant amounts, up to a maximum concentration of 20 μg/mL of bound AFB_1_. Both viable and non-viable cells are effective aflatoxin binders and non-viable cells bound higher levels of the toxin than viable cells (Shetty et al., 2007). *Lactiplantibacillus plantarum* is a facultative heterofermentative LAB, 17 out of 33 LAB from plants showed removal of ZEN from the liquid medium, with *L. plantarum* BCC 47723 isolated from wild spider flower pickle (Pag-sian-dorng) showing the highest removal (23%) (Adunphatcharaphon et al., 2021).

## 4 Conclusion

There are approximately 400 known mycotoxins that contaminate food, feed, fruits, and crops and cause a range of physiological toxicity in humans and other animals. For safety purposes, many countries have imposed mandatory thresholds for mycotoxin levels. The most important step in controlling mycotoxins is prevention, i.e., preventing fungal contamination and the production of mycotoxins. However, mycotoxin contamination often cannot be prevented completely throughout the growth, storage, and transportation of crops as well as the production, processing and preservation of food; thus, a detoxification method must be developed to treat mycotoxin-contaminated foods (raw materials) and eliminate or reduce toxicity to humans and animals. Mycotoxins can be detoxified by physical, chemical and biological methods. Biological enzymatic methods are advantageous over physical and chemical methods in terms of their selectivity, safety and environmental friendliness. However, these methods face the following challenges: (1) enzymatic detoxification mostly reduces the toxicity of mycotoxins, as the products of enzyme degradation are often less toxic but still exhibit physiological toxicity; (2) the processing and use of fungal-contaminated raw materials are sometimes affected after treatment with enzymatic detoxification processes; (3) at present, only a few types of mycotoxins and their degradation enzymes and degradation mechanisms have been thoroughly studied, and some mycotoxin degradation enzymes or degradation mechanisms remain unclear; (4) information on the structures of mycotoxin degradation products and their physiological toxicities is lacking, and more research is needed to improve knowledge of mycotoxin degradation products; and (5) relevant mycotoxin detection technology and screening, optimization and safety assessment of biodegradation enzymes still need to be improved. In this paper, we reviewed the main enzymes used to detoxify mycotoxins and the mechanisms underlying detoxification. Based on these findings, several aspects of the native enzymes used for mycotoxin detoxification, including their instability, low activity, and inhibitory activity against products or substrates, should be improved. In the past 10 years, genetic engineering technology has been widely used for heterologous mycotoxin detoxification enzyme expression and engineering of modifications to enhance the detoxification effect of enzymes to expand their application. Additionally, studying enzyme immobilization has become an important method for improving the efficiency of enzymes in the application of detoxification enzymes. Studies on the structural biology of existing detoxification enzymes have revealed the mechanism by which enzymes detoxify mycotoxins, and potential detoxification enzymes can be better explored through the inclusion of bioinformatics. As a green detoxification method, the technology used for the enzymatic detoxification of mycotoxins is maturing; in addition, the industrialization process is accelerating, and the application fields are expanding. In the future, research on the enzymatic detoxification of mycotoxins should focus on the development of high-efficiency enzymes, the development of multienzyme cascades and synergistic degradation systems, and the development of detoxification enzymes and production systems that can be used directly in foods and feeds, are particularly important.

## Author contributions

ML: Data curation, Formal analysis, Investigation, Validation, Writing – original draft, Writing – review & editing. XZ: Investigation, Resources, Software, Supervision, Validation, Writing – original draft, Writing – review & editing. HL: Data curation, Formal analysis, Investigation, Writing – original draft. YZ: Data curation, Formal analysis, Investigation, Writing – review & editing. WX: Conceptualization, Supervision, Visualization, Writing – review & editing. WF: Supervision, Visualization, Writing – review & editing. PS: Conceptualization, Funding acquisition, Project administration, Supervision, Validation, Visualization, Writing – review & editing.
